# Supplementary material for: Rethinking simultaneous suppression in visual cortex via compressive spatiotemporal population receptive fields
Source: Nat Commun. 2024 Aug 11;15:6885. doi: 10.1038/s41467-024-51243-7 (PMC11317513; doi:10.1038/s41467-024-51243-7)
Supplement: Supplementary file 1 — Supplementary Information [file 41467_2024_51243_MOESM1_ESM.pdf]

Supplementary Material

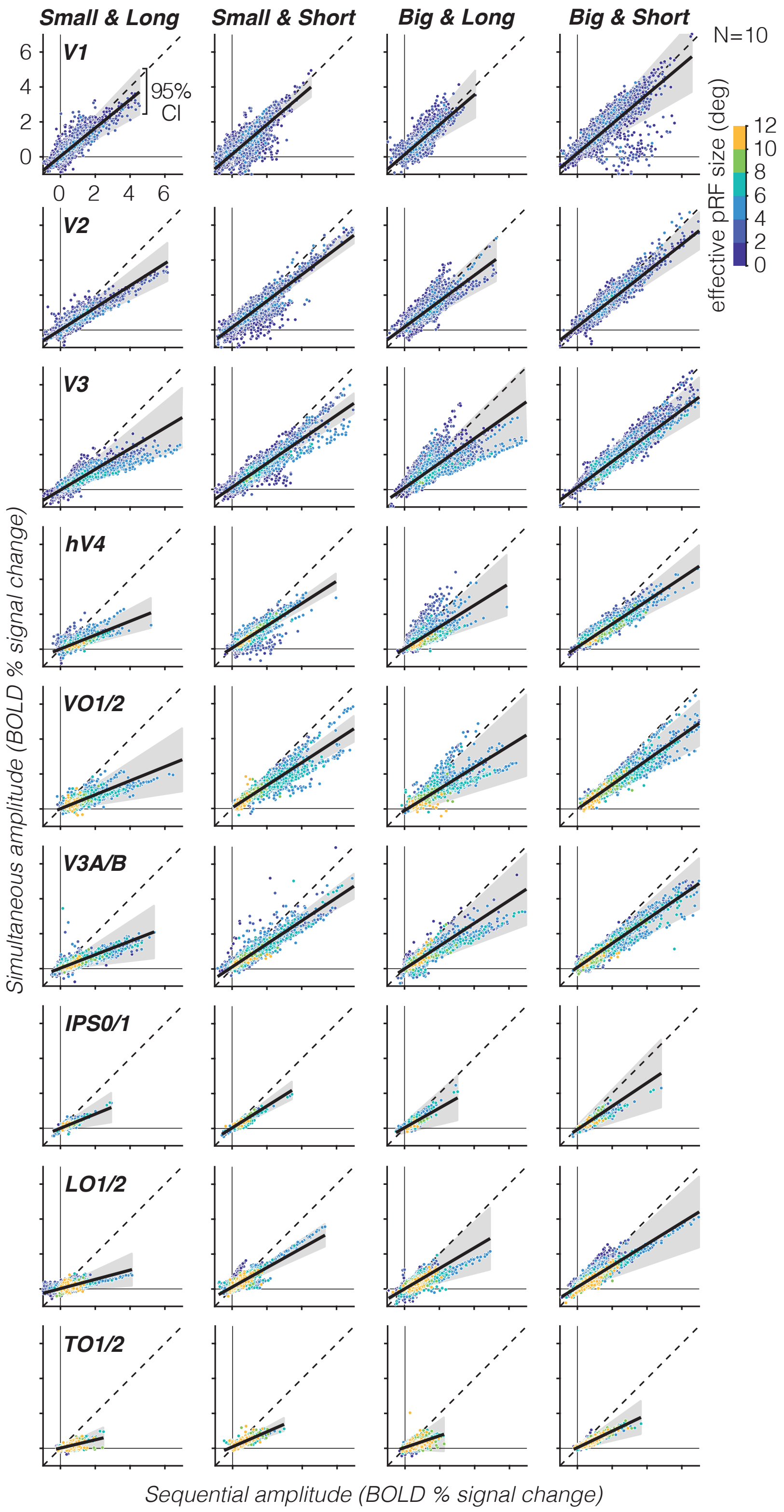

Supplementary Figure 1. Average sequential vs simultaneous BOLD amplitude of individual voxels for all stimulus conditions. Each point is a voxel. Voxels are colored by effective pRF size estimated from independent retinotopy data in six 2º non-overlapping bins. Each panel shows data of all 10 participants. *Black solid line:* Average LMM slope across participants. *Shaded area:* 95%-confidence interval (CI) across participants. *Dashed line*: Identity line, no suppression.

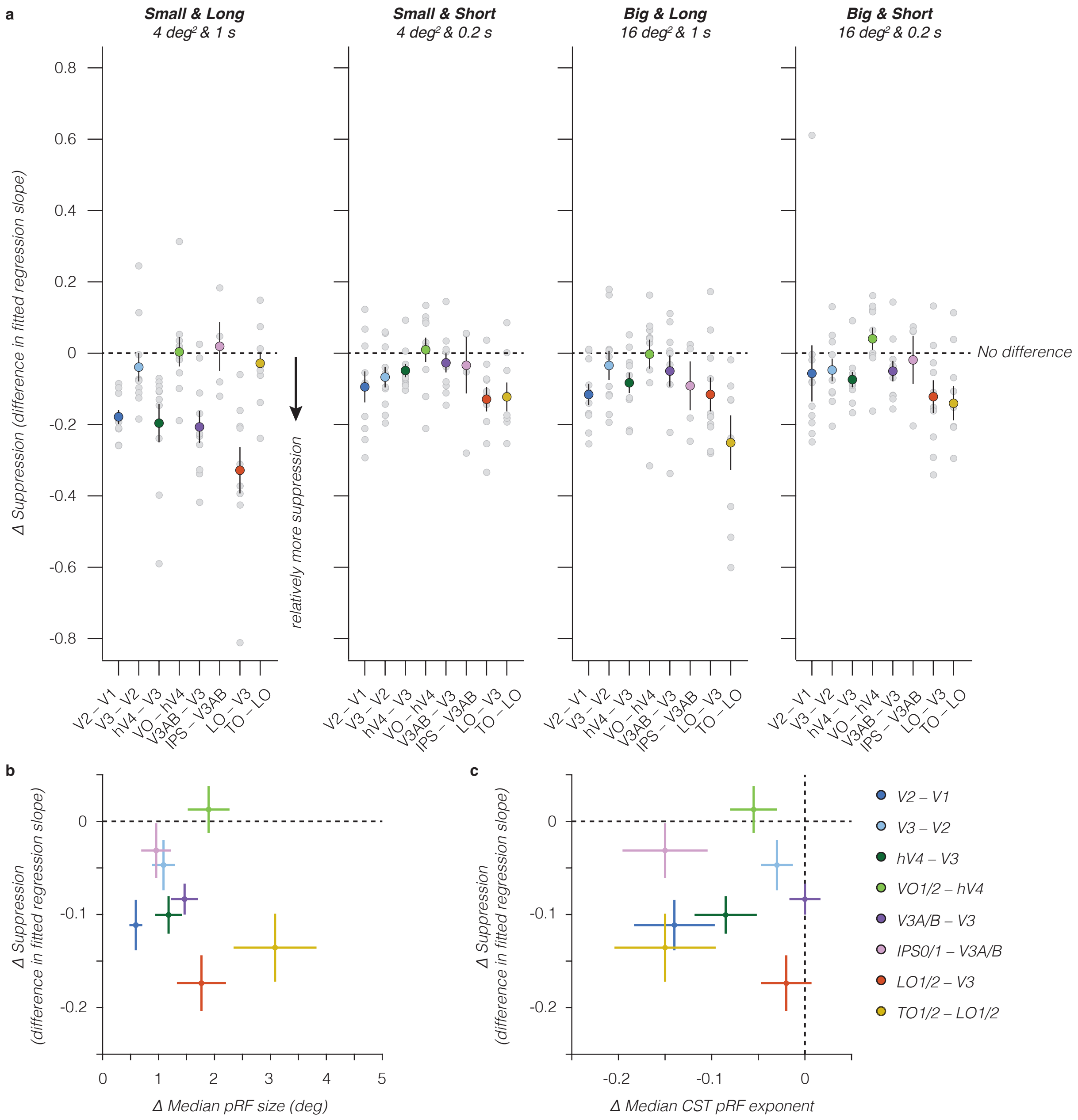

Supplementary Figure 2. Accumulation of simultaneous suppression up the visual hierarchy. a Difference in suppression levels between pairs of consecutive visual areas for each stimulus condition. Difference (Δ) in suppression values are derived by subtracting participants' regression slopes of the earlier visual area from the subsequent visual area (e.g., V2–V1). *Large colored dots:* Group average of 10 participant's difference in suppression for each pair of visual areas. Zero indicates no difference in suppression. Negative values indicate more suppression in the later than earlier visual area. Positive values indicate more suppression in the earlier than later visual area. We find that the difference in simultaneous suppression levels across consecutive visual areas varies by stimulus condition and that there is neither a monotonic increase nor plateauing of suppression across visual processing hierarchies. For example, for long duration stimuli, we observe a "sawtooth" pattern where suppression increases more from V1 to V2 than V2 to V3, and more from V3 to hV4 than hV4 to VO1/2 in the ventral stream. This suggests no monotonic increase in suppression from mid-level area hV4 to higher-level visual areas VO1/2. *Light gray dots:* Individual participant differences in suppression. *Dark blue:* V2–V1. *Light blue:* V3–V2, *Dark green:* hV4–V3. *Light green:* VO1/2–-hV4. *Purple:*

V3A/B–V3. *Pink:* IPS0/1–V3A/B. *Red:* LO1/2–V3. *Yellow:* TO1/2–LO1/2. *Error bars:* SEM across 10 participants. **b** Difference in suppression levels vs median pRF size. **c** Difference in suppression levels vs median CST pRF exponent. In panel b and c, difference in suppression values are averaged across stimulus conditions within a participant, then computing the difference in suppression between visual areas. Differences in pRF size and pRF exponent are derived by first subtracting median pRF size (or CST exponent) of the earlier visual area from the later visual area within participants, then averaging differences values across 10 participants for each area pair (dots). *Error bars*: Standard error across the mean (SEM) across 10 participants. *Dashed line:* No difference in suppression slopes. Overall, we find no clear relation between differences in suppression levels with the difference in pRF size (b) or spatiotemporal compression (c) between paired visual areas.

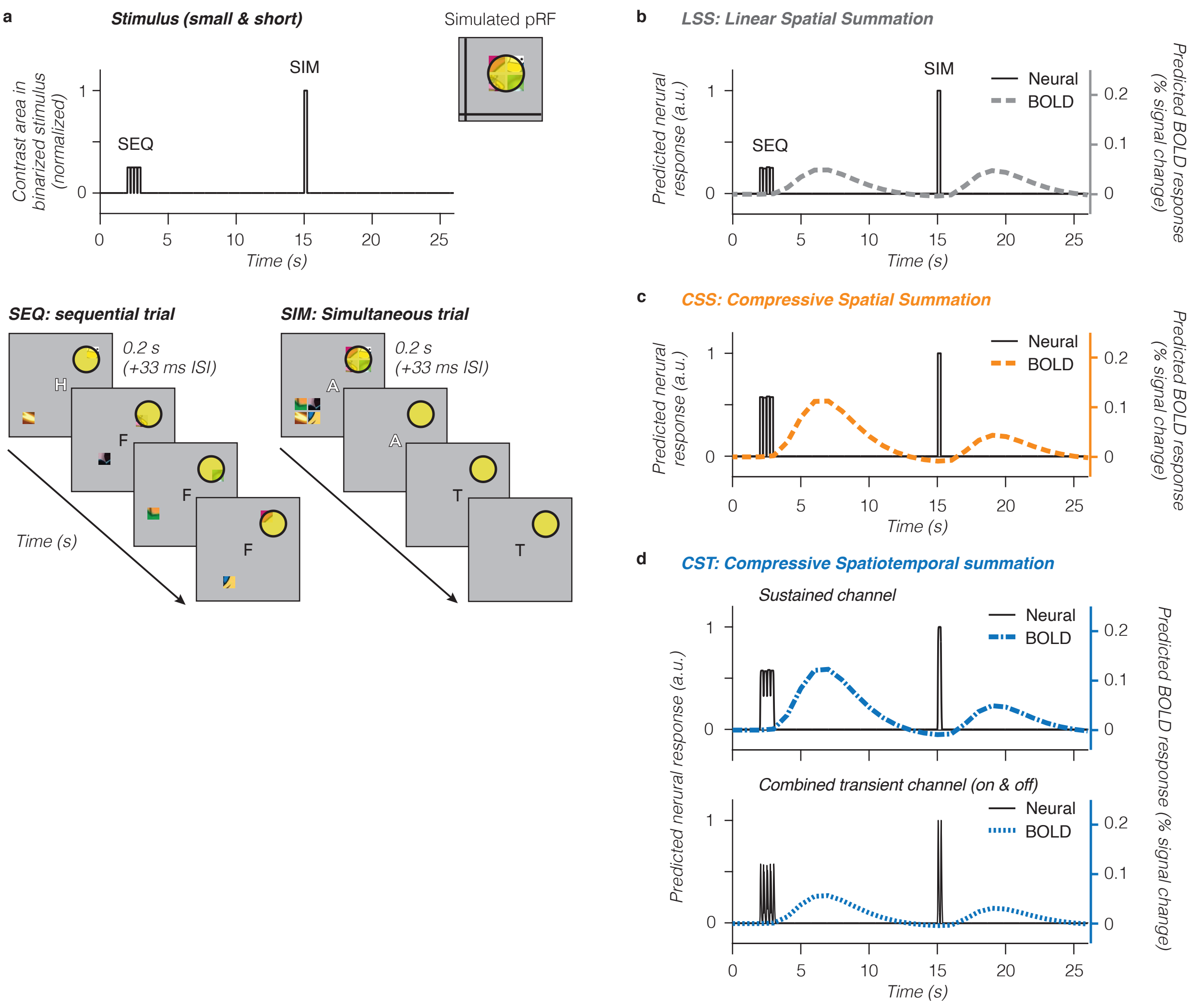

Supplementary Figure 3. Simulated pRF model predictions for a voxel with a pRF overlapping the four stimuli in the upper visual field for single sequential trial followed by a simultaneous trial. Each model simulation uses a large pRF (see inset in panel a) that overlaps four small squares. In the simulation, we used 0.2 s trials (short timing). **a** Stimulus time series. The stimulus' visual extent is represented as the total contrast area in a binarized stimulus frame, where pixels are summed across space for each time point and normalized to set the maximum contrast area to 1. Because each trial has 4 squares per quadrant, the contrast area for each square in the sequential trial (SEQ) is a fourth of the area when all squares are shown simultaneously (SIM). **b-d** PRF model predictions. *Black lines & left y-axis:* predicted neural response. *Colored lines & right y-axis:* predicted BOLD response. *a.u.:* arbitrary units. **b** Linear spatial summation (LSS) pRF prediction (dashed gray). The LSS model sums stimulus input linearly over time and space. This linearity, combined with individual squares in simultaneous and sequential trials being matched in duration and location relative to the pRF, results in the LSS model predicting no simultaneous suppression. **c** Compressive spatial summation (CSS) pRF prediction (dashed orange). Due to the compressive static nonlinearity, the CSS model predicts simultaneous suppression when multiple squares (simultaneously) overlap with the pRF than when a single square (sequentially) overlaps the pRF. The CSS pRF model sums linearly in time and as the overall stimulus duration is similar in our experiment between the blocks of short and long conditions, it will not predict differences in response amplitude for short vs long stimulus presentation timings (not shown in this simulation). **d** Compressive spatiotemporal summation (CST) pRF prediction (blue). *Blue dot-dashed:* sustained spatiotemporal channel. *Blue dashed:* combined on- and off-transient spatiotemporal channel. By explicitly encoding neural temporal transients in milliseconds, the CST model predicts larger BOLD responses for many

visual transients (SEQ) vs a few transients (SIM). The static nonlinearity produces additional subadditive spatiotemporal summation for both sustained and transient channels, including spatial subadditivity when multiple squares overlap the pRF. Consequently, both CST channels generate larger responses for sequential than simultaneous presentations, and predict different responses for the short and long conditions (not shown in simulation), which vary by a factor of 4 in number of transients (Fig 1b).

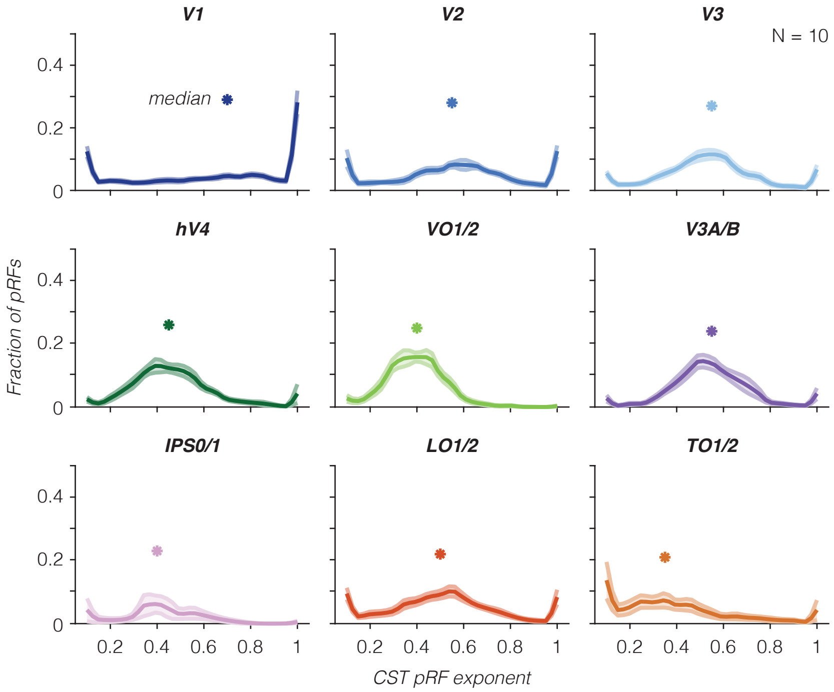

Supplementary Figure 4. Average CST pRF exponent parameter distributions. Distributions are computed by first resampling participants' data 1000 times per visual area, then averaging distributions across 10 participants. Both group average (line) and SEM (shaded area) of each visual area distribution are then upsampled 2x. *Asterisks:* median CST exponent value.

Eye movement analysis. Raw horizontal and vertical gaze position (deg) and velocity (deg/s) time series of 5 participants during SEQ-SIM fMRI experiment were preprocessed as follows. First, we removed time points occurring within -100 to 100 ms of blinks. Second, given large amounts of spatial noise, we used the Identification by Two-Means Clustering algorithm[1] to label robust fixation periods and their visual field location. If gaze locations jumped between two means due to noise, we recentered data to a single mean. Third, we removed time points (and surrounding 2 ms) if they had (i) a velocity larger than a typical saccade up to 8º (400 deg/s)[2], (ii) an absolute gaze location that extended beyond the stimulus display (radius = 10º), or (iii) a gaze position SD 2.5x larger than SD across horizontal and vertical time series. We excluded 7 runs with < 20% data, resulting in 32 runs total. We visualized participant's median and kernel density of gaze location across runs in visual space.

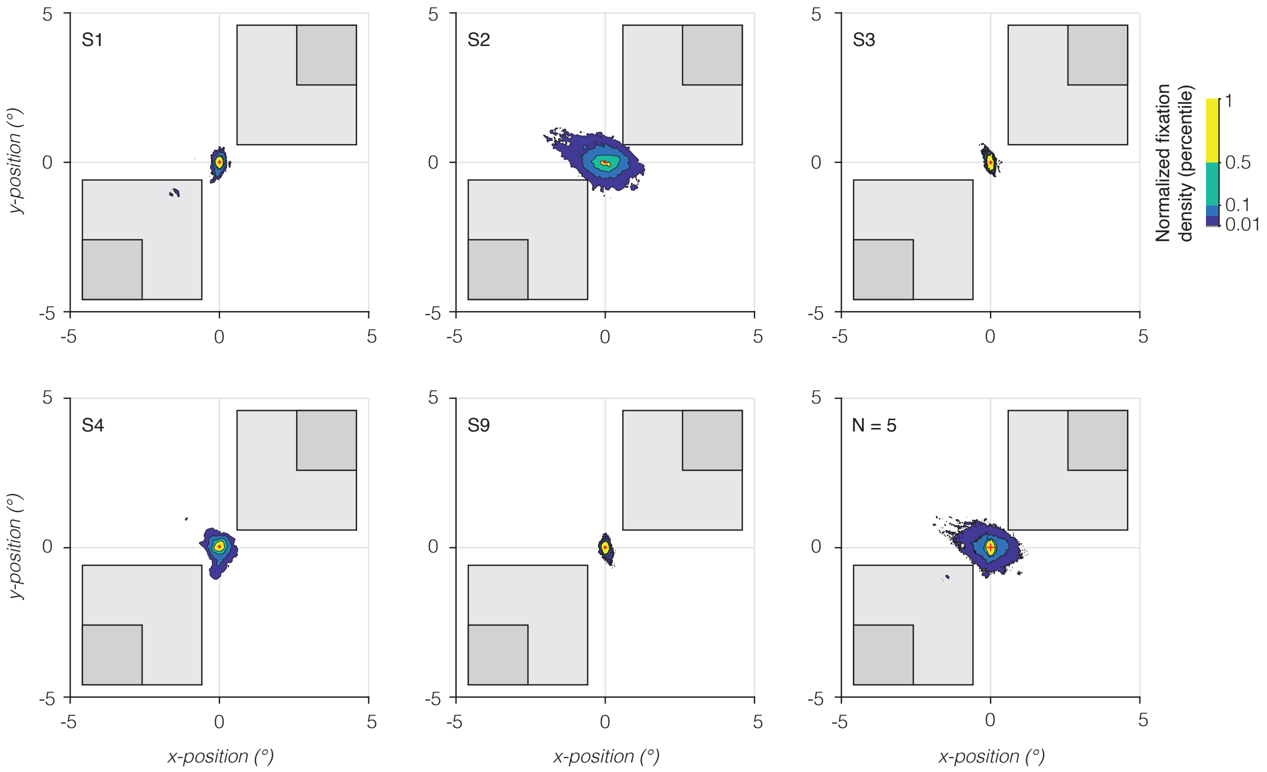

Supplementary Figure 5. Eye fixation locations during SEQ-SIM experiment. Normalized fixation density is shown for 5 participants (S1, S2, S3, S4, S9) and across all participants (N=5). *Red cross:* Median gaze location across runs. *Contour lines*: Density at 1st, 10th, 50th, 100th percentile, correspond to dark blue, light blue, green, and yellow sections. *Light gray squares:* Outlined location of large squares closest to fixation ([x,y]=[0,0]). *Dark gray squares:* Outlined location of small squares closest to fixation.

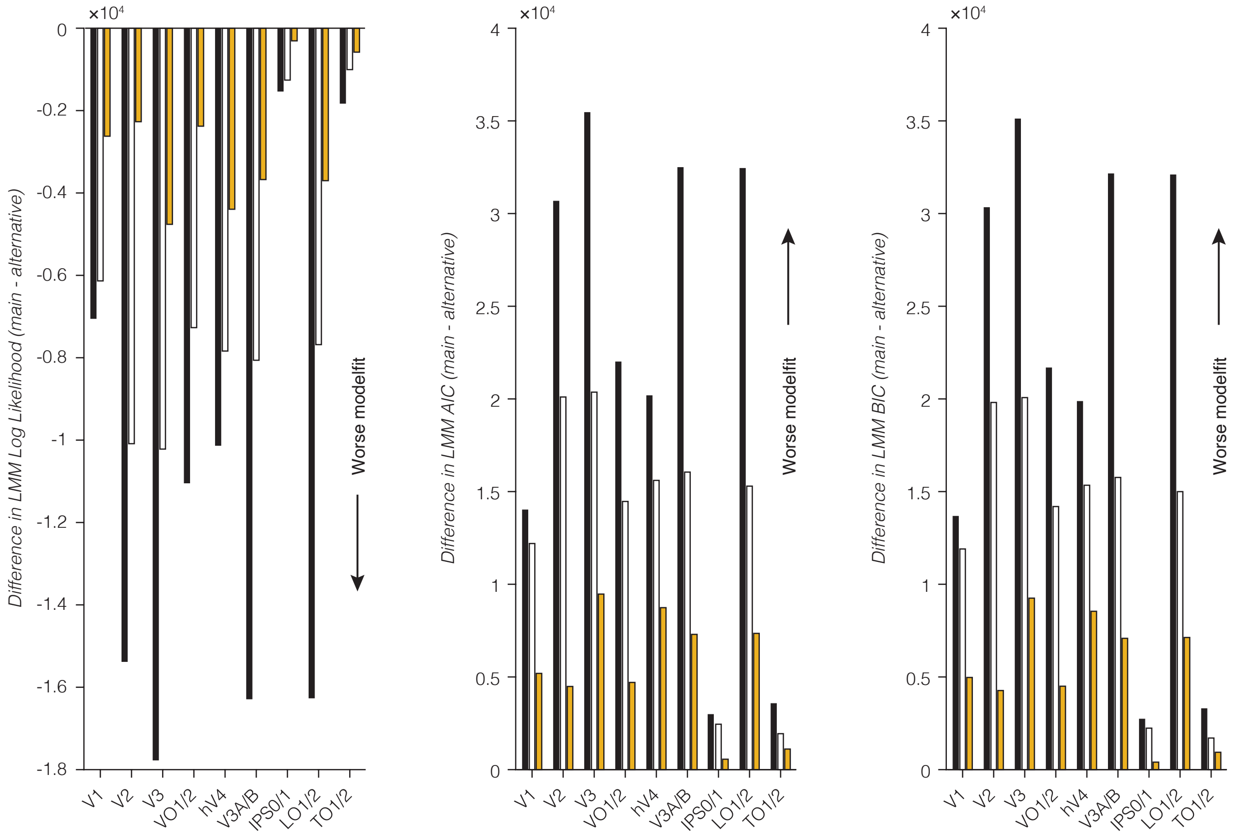

Main LMM: mean SIM ampl ~ 1 + mean SEQ ampl × Condition + (1 + mean SEQ ampl × Condition | Participant)

LMM alternatives

- LMM 1: mean SIM ampl ~ 1 + mean SEQ ampl + (1 | Participant)
- LMM 2: mean SIM ampl ~ 1 + mean SEQ ampl × Condition + (1 | Participant)
- LMM 3: mean SIM ampl ~ 1 + mean SEQ ampl × Condition + (Condition | Participant)

Supplementary Figure 6. Comparison of linear mixed models (LMMs). For each model comparison metric, we computed the difference between main LMM and alternative LMM. The main LMM fits the data better than all alternative LMMs, for each visual area, on each metric. *Right:* Difference in LMM log likelihood. *Middle:* Difference in LMM AIC. *Left:* Difference in LMM BIC. The main LMM uses a fixed intercept and slope for mean sequential (SEQ) amplitude as a function of stimulus condition and allows for random participant intercept and slope per stimulus condition. *Black bar:* Difference between main LMM and alternative LMM 1, which has a fixed intercept and slope for mean sequential (SEQ) amplitude and allows one random slope per participant (N=10). *White bar:* Difference between main LMM and alternative LMM 2, which has a fixed intercept and slope for mean sequential (SEQ) amplitude as a function of stimulus condition and allows one random slope per participant. *Yellow bar:* Difference between main LMM and alternative LMM 3, which as a fixed intercept and slope for mean sequential (SEQ) amplitude as a function of stimulus condition and allows a random slope per participant, per condition.

Supplementary Table 1. Summary of suppression slopes for 9 visual areas and 4 stimulus conditions. Data are scale factors and have arbitrary units. Data are from 10 participants, except for IPS0/1 (4 participants) and TO1/2 (8 participants). M: mean. SE: standard error.

	Stimulus condition							
	Small & Short		Small & Long		Big & Short		Big & Long	
Visual area	*M*	*SE*	*M*	*SE*	*M*	*SE*	*M*	*SE*
V1	0.85	0.057	0.81	0.023	0.84	0.070	0.85	0.036
V2	0.75	0.028	0.63	0.023	0.78	0.037	0.78	0.055
V3	0.67	0.040	0.59	0.054	0.74	0.039	0.70	0.076
hV4	0.64	0.037	0.40	0.029	0.66	0.040	0.62	0.080
VO1/2	0.65	0.051	0.40	0.034	0.70	0.042	0.62	0.070
V3A/B	0.66	0.051	0.39	0.043	0.67	0.035	0.65	0.059
IPS0/1	0.63	0.061	0.41	0.054	0.67	0.10	0.56	0.056
LO1/2	0.56	0.057	0.27	0.041	0.61	0.051	0.59	0.064
TO1/2	0.43	0.057	0.24	0.043	0.47	0.019	0.47	0.11

Supplementary Table 2. Post-hoc comparisons of suppression slopes between two stimulus conditions, for each visual area. Mean slope difference between conditions (C1 – C2), standard error (SE) and 95%-confidence intervals ($CI_{95\%}$). Data are from 10 participants, except for IPS0/1 (N=4) and TO1/2 (N=8). Student's t-test (two-sided); *p*-values are Bonferroni-corrected for multiple comparisons. *ns:* not significant.

Visual area	Condition 1	Condition 2	C1 – C2	SE	$CI_{95\%}$ Lower	$CI_{95\%}$ Upper	*p*
V1	Short & big	Long & big	-0.013	0.076	-0.21	0.19	ns
	Short & big	Short & small	-0.0071	0.076	-0.21	0.19	ns
	Short & big	Long & small	0.030	0.076	-0.17	0.23	ns
	Long & big	Short & small	0.0054	0.076	-0.20	0.22	ns
	Long & big	Long & small	0.042	0.076	-0.19	0.24	ns
	Short & small	Long & small	0.037	0.076	-0.16	0.24	ns
V2	Short & big	Long & big	0.047	0.076	-0.15	0.25	ns
	Short & big	Short & small	0.032	0.076	-0.17	0.23	ns
	Short & big	Long & small	0.15	0.076	-0.049	0.35	ns
	Long & big	Short & small	-0.016	0.076	-0.22	0.18	ns
	Long & big	Long & small	0.12	0.076	-0.096	0.31	ns
	Short & small	Long & small	0.12	0.076	-0.080	0.32	ns
V3	Short & big	Long & big	0.034	0.076	-0.17	0.23	ns
	Short & big	Short & small	0.051	0.076	-0.15	0.25	ns
	Short & big	Long & small	0.14	0.076	-0.057	0.34	ns
	Long & big	Short & small	0.017	0.076	-0.18	0.22	ns
	Long & big	Long & small	0.11	0.076	-0.091	0.31	ns
	Short & small	Long & small	0.093	0.076	-0.11	0.29	ns
hV4	Short & big	Long & big	0.043	0.076	-0.16	0.24	ns
	Short & big	Short & small	0.025	0.076	-0.18	0.23	ns
	Short & big	Long & small	0.27	0.076	0.066	0.47	3.0×10^{-3}
	Long & big	Short & small	-0.018	0.076	-0.22	0.18	ns
	Long & big	Long & small	0.22	0.076	0.022	0.42	2.1×10^{-2}
	Short & small	Long & small	0.24	0.076	0.040	0.44	9.7×10^{-3}
VO1/2	Short & big	Long & big	0.086	0.076	-0.11	0.29	ns
	Short & big	Short & small	0.056	0.076	-0.14	0.26	ns
	Short & big	Long & small	0.30	0.076	0.10	0.50	4.8×10^{-4}
	Long & big	Short & small	-0.030	0.076	-0.23	0.17	ns
	Long & big	Long & small	0.22	0.076	0.016	0.42	2.7×10^{-2}
	Short & small	Long & small	0.25	0.076	0.046	0.45	7.5×10^{-3}
V3A/B	Short & big	Long & big	0.033	0.076	-0.17	0.23	ns
	Short & big	Short & small	0.027	0.076	-0.17	0.23	ns
	Short & big	Long & small	0.30	0.076	0.099	0.50	5.4×10^{-4}
	Long & big	Short & small	-0.0061	0.076	-0.21	0.19	ns
	Long & big	Long & small	0.27	0.076	0.066	0.47	2.9×10^{-3}
	Short & small	Long & small	0.27	0.076	0.072	0.47	2.2×10^{-3}
IPS0/1	Short & big	Long & big	0.12	0.12	-0.21	0.42	ns
	Short & big	Short & small	0.042	0.12	-0.27	0.36	ns
	Short & big	Long & small	0.26	0.12	-0.056	0.58	ns
	Long & big	Short & small	-0.064	0.12	-0.38	0.25	ns
	Long & big	Long & small	0.16	0.12	-0.16	0.47	ns
	Short & small	Long & small	0.22	0.12	-0.098	0.54	ns
LO1/2	Short & big	Long & big	0.027	0.076	-0.17	0.23	ns
	Short & big	Short & small	0.058	0.076	-0.14	0.26	ns
	Short & big	Long & small	0.35	0.076	0.15	0.55	3.3×10^{-5}
	Long & big	Short & small	0.030	0.076	-0.17	0.23	ns
	Long & big	Long & small	0.32	0.076	0.12	0.52	1.6×10^{-4}
	Short & small	Long & small	0.29	0.076	0.091	0.49	8.2×10^{-4}
TO1/2	Short & big	Long & big	0.14	0.084	-0.086	0.36	ns
	Short & big	Short & small	0.040	0.084	-0.18	0.26	ns
	Short & big	Long & small	0.24	0.084	0.013	0.46	3.1×10^{-2}
	Long & big	Short & small	−0.098	0.084	-0.32	0.13	ns
	Long & big	Long & small	0.10	0.084	-0.12	0.32	ns
	Short & small	Long & small	0.20	0.084	-0.027	0.42	ns

Supplementary Table 3. Post-hoc comparisons of pRF model performance. Mean difference in pRF model performance (M1 – M2), standard error (SE) and 95%-confidence intervals ($CI_{95\%}$) are in units of percent cross-validated variance explained (cv-R^2) and correspond to violin plots in Fig 6c. Data are from 10 participants, except for IPS0/1 (N=4) and TO1/2 (N=8). Student's t-test (two-sided); *p*-values are Bonferroni-corrected for multiple comparisons. *ns:* not significant.

Visual area	Model 1	Model 2	M1 – M2	SE	$CI_{95\%}$ Lower	$CI_{95\%}$ Upper	*p*
V1	CSS	LSS	−1.38	0.240	−1.95	−.804	2.7×10^{-8}
	CST	CSS	6.54	0.240	5.97	7.12	1.6×10^{-163}
	CST	LSS	5.17	0.239	4.59	5.74	1.1×10^{-102}
V2	CSS	LSS	−0.407	0.239	−0.98	0.164	ns
	CST	CSS	9.33	0.239	8.76	9.90	1.0×10^{-304}
	CST	LSS	8.92	0.239	8.35	9.49	3.4×10^{-304}
V3	CSS	LSS	0.250	0.238	−0.318	0.819	ns
	CST	CSS	10.42	0.238	9.85	11.0	1.0×10^{-304}
	CST	LSS	10.67	0.238	10.10	11.2	1.0×10^{-304}
hV4	CSS	LSS	3.21	0.316	2.45	3.97	$7. 9\times10^{-24}$
	CST	CSS	8.02	0.316	7.26	8.77	1.2×10^{-141}
	CST	LSS	11.2	0.316	10.5	12.0	9.8×10^{-276}
VO1/2	CSS	LSS	4.92	0.376	4.02	5.82	1.3×10^{-38}
	CST	CSS	8.88	0.376	7.98	9.78	2.4×10^{-122}
	CST	LSS	13.80	0.376	12.90	14.7	1.1×10^{-292}
V3A/B	CSS	LSS	1.02	0.264	0.386	1.65	3.4×10^{-4}
	CST	CSS	8.63	0.264	8.00	9.26	8.2×10^{-234}
	CST	LSS	9.65	0.264	9.02	10.3	1.7×10^{-291}
IPS0/1	CSS	LSS	2.77	0.776	0.912	4.63	1.1×10^{-3}
	CST	CSS	6.77	0.776	4.91	8.62	8.1×10^{-18}
	CST	LSS	9.53	0.776	7.68	11.4	3.0×10^{-34}
LO1/2	CSS	LSS	1.16	0.231	0.606	1.71	1.6×10^{-06}
	CST	CSS	4.44	0.231	3.89	5.00	6.7×10^{-82}
	CST	LSS	5.60	0.231	5.05	6.15	2.9×10^{-129}
TO1/2	CSS	LSS	3.95	0.563	2.60	5.30	6.6×10^{-12}
	CST	CSS	3.34	0.563	1.99	4.69	8.8×10^{-9}
	CST	LSS	7.29	0.563	5.95	8.64	6.2×10^{-38}

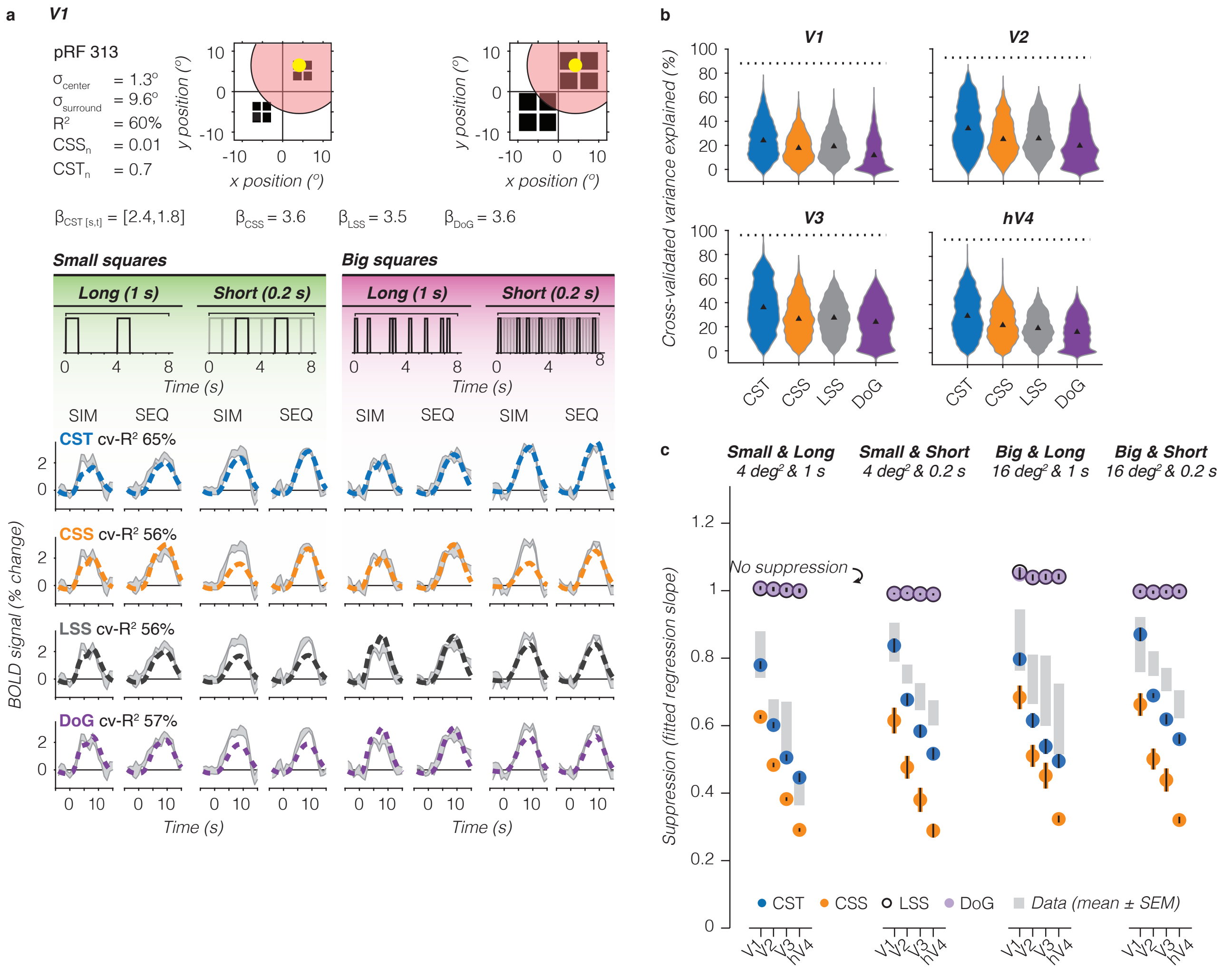

Supplementary Figure 7. Simulated Difference of Gaussians pRF model performs similarly to the LSS pRF model. a V1 example voxel. *Gray shaded area:* Average ± SEM voxel time series, repeated for each row. PRF model fits are shown in dashed lines. *Dark Blue:* Compressive spatiotemporal summation model with fixed voxel parameters (CST, first row). *Orange:* Compressive spatial summation model (CSS, second row). *Black:* Linear spatial summation model (LSS, third row). *Purple:* Difference of Gaussians model (DoG, fourth row). DoG pRF surround size is based on each visual area's average pRF center/surround ratio reported by Aqil *et al.* (2021)[3]. **b** Distribution of voxel-level cross-validated variance explained for each pRF model. *Triangle:* median. *Dotted line:* noise ceiling computed from voxels' maximum split-half reliability across participants. CST (blue), CSS (orange), and LSS (gray) violin plots are the same as in Fig 6c. *Purple*: DoG. Each participant's data is resampled 1000 times for each visual area. **c** Model-based prediction of simultaneous suppression vs observed simultaneous suppresion. Panel shows the same observed and predicted CST, CSS, and LSS suppression levels as Fig 7, with the addition of predicted suppression levels by the simulated DoG pRFs (semi-saturated filled purple circles). Model-based points and errorbars show average and SEM across 10 participants.

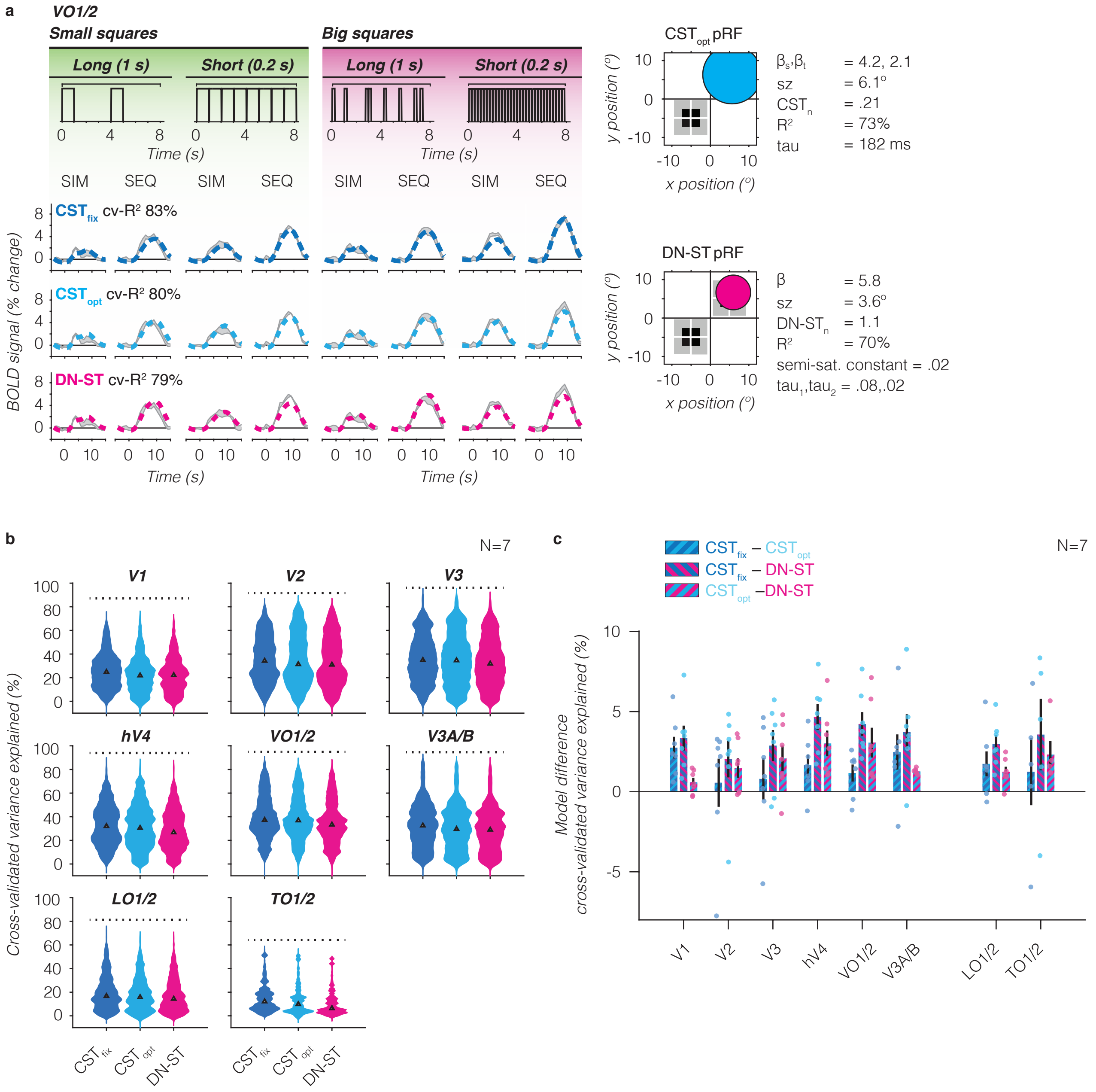

Supplementary Figure 8. Comparison of fixed vs optimized CST and DN-ST pRF model performance. **a** VO1/2 example voxel. *Gray shaded area:* Average ± SEM voxel time series. CST_{fix} data are from the same voxel as in Fig 6b. CST_{opt} and DN-ST example voxels show corresponding time series to CST_{fix} time series from the same participant (S3), but have different pRF parameters from Kim *et al.* (2024)[4]. PRF model fits are shown in dashed lines. *Dark Blue:* Compressive spatiotemporal summation model with fixed voxel parameters (CST_{fix}, top row). *Cyan:* Compressive spatiotemporal summation model with optimized voxel parameters (CST_{opt}, middle row). *Pink:* Divisive normalization spatiotemporal summation model with optimized voxel parameters (DN-ST, bottom row). **b** Distribution of voxel-level cross-validated variance explained for each pRF model. Each violin plot contains data from the 7 overlapping participants with Kim *et al.* (2024). *Triangle:* Median. *Dotted line:* Noise ceiling computed from voxel's maximum split-half reliability across participants. *Blue:* CST_{fix}. *Cyan*: CST_{opt}. *Pink:* DN-ST. Each participant's data is resampled 1000x for each visual area. A two-way repeated measures ANOVA revealed significant effects of pRF model ($F(2) = 1.1\text{x}10^2$, $p = 6.3\text{x}10^{-47}$)

and ROI ($F(7) = 1.3\text{x}10^3$, $p = 10^{-48}$) on cv-R^2, with CST models outperforming DN-ST on average by about 3%, as well as a significant interaction between pRF model and ROI ($F(2,7) = 5.0$, $p = 1.6\text{x}10^{-9}$). Post-hoc Bonferroni-corrected t-tests revealed that both CST models significantly explain more cv-R^2 than the DN-ST model in all visual areas, except for CST_{opt} in TO1/2. In addition, the CST_{fix} model explains significantly more cv-R^2 than the CST_{opt} in several visual areas (V1, hV4, V3A/B, LO1/2, and TO1/2). **c** Pairwise model comparison for each visual area. *Bars:* Group average voxelwise difference in cv-R^2 between two pRF models. *Error bars:* SEM across 7 participants. *Individual dots:* Average difference for each participant. *Dark blue–cyan:* CST_{fix} vs CST_{opt}. *Dark blue–pink:* CST_{fix} vs DN-ST. *Cyan–pink:* CST_{opt} vs DN-ST.

Supplementary Table 4. ***Post-hoc comparisons of spatiotemporal pRF model performance.*** Mean difference in pRF model difference (M1 – M2), standard error, and 95%-confidence intervals are in units of percent cross-validated variance explained (cv-R^2) and correspond to violin plots in Supplementary Fig 8b. Data are from 7 overlapping participants with Kim *et al.* (2024)[4]. IPS0/1 results are removed as only two participants contributed data for these visual areas. Student's t-test (two-sided); *p*-values are Bonferroni-corrected for multiple comparisons. *ns*: not significant.

Visual area	Model 1	Model 2	M1 – M2	SE	$CI_{95\%}$ Lower	$CI_{95\%}$ Upper	*p*
V1	CST_{fix}	CST_{opt}	2.35	0.550	1.03	3.67	5.9×10^{-5}
	CST_{fix}	DN-ST	2.89	0.550	1.58	4.21	4.4×10^{-7}
	CST_{opt}	DN-ST	0.54	0.550	−0.775	1.86	ns
V2	CST_{fix}	CST_{opt}	-0.16	0.428	−1.18	0.870	ns
	CST_{fix}	DN-ST	1.24	0.428	0.209	2.26	1.2×10^{-2}
	CST_{opt}	DN-ST	1.39	0.429	0.363	2.42	3.6×10^{-3}
V3	CST_{fix}	CST_{opt}	0.95	0.436	−0.100	1.99	ns
	CST_{fix}	DN-ST	3.00	0.436	1.96	4.05	2.0×10^{-11}
	CST_{opt}	DN-ST	2.06	0.436	1.01	3.10	7.6×10^{-6}
hV4	CST_{fix}	CST_{opt}	1.79	0.636	0.27	3.31	1.4×10^{-2}
	CST_{fix}	DN-ST	5.08	0.636	3.56	6.60	4.2×10^{-15}
	CST_{opt}	DN-ST	3.23	0.636	1.76	4.81	7.1×10^{-7}
VO1/2	CST_{fix}	CST_{opt}	0.92	0.630	−0.584	2.43	ns
	CST_{fix}	DN-ST	4.56	0.630	3.05	6.07	1.4×10^{-12}
	CST_{opt}	DN-ST	3.63	0.631	2.12	5.15	2.6×10^{-8}
V3A/B	CST_{fix}	CST_{opt}	3.34	0.510	2.12	4.56	1.6×10^{-10}
	CST_{fix}	DN-ST	4.58	0.510	3.36	5.80	7.4×10^{-19}
	CST_{opt}	DN-ST	1.24	0.511	0.0173	2.46	4.6×10^{-2}
LO1/2	CST_{fix}	CST_{opt}	1.35	0.526	0.0881	2.61	3.1×10^{-2}
	CST_{fix}	DN-ST	2.88	0.526	1.62	4.14	1.3×10^{-7}
	CST_{opt}	DN-ST	1.53	0.527	0.272	2.79	1.1×10^{-2}
TO1/2	CST_{fix}	CST_{opt}	4.40	1.56	0.652	8.14	1.5×10^{-2}
	CST_{fix}	DN-ST	6.61	1.56	2.86	10.4	7.2×10^{-5}
	CST_{opt}	DN-ST	2.21	1.57	−1.55	5.98	ns

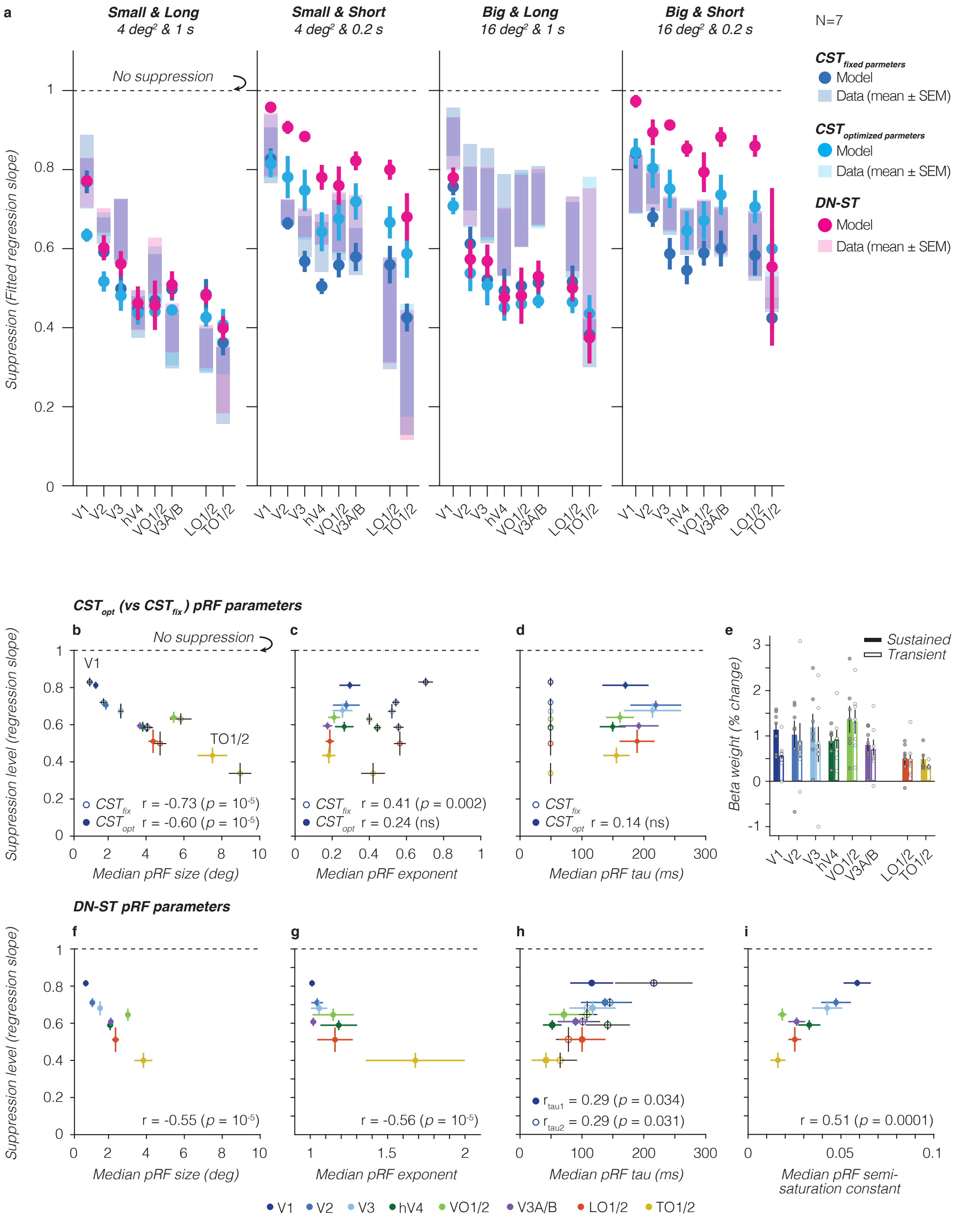

Supplementary Figure 9. Comparison of compressive spatiotemporal pRF models. Suppression levels and pRF parameters are from voxels overlapping SEQ-SIM squares for the 7 participants overlapping with Kim

et al. (2024)[4]. In all panels: *dots/bars* show average across 7 participants. *Error bars:* SEM across 7 participants. IPS0/1 results are removed as only two participants contributed data for these visual areas. **a** CST_{fix}, CST_{opt}, and DN-ST pRF model-based predictions of simultaneous suppression. Shaded *dark blue bars:* Average ± SEM observed suppression levels in voxels with CST_{fix} pRFs. *Shaded cyan bars:* Average ± SEM observed suppression levels in voxels with CST_{opt} pRFs. *Shaded pink bars:* Average ± SEM observed suppression levels in voxels with DN-ST pRFs. *Dark blue circles*: Average ± SEM predicted simultaneous suppression by CST_{fix} pRFs. *Cyan filled circles:* Average ± SEM predicted simultaneous suppression by CST_{opt} pRFs. *Pink filled circles:* Average ± SEM predicted simultaneous suppression by DN-ST pRFs. The DN-ST pRFs tends to underpredict simultaneous suppression levels for short stimulus durations, which may be due to less accurate temporal parameter recovery for the DN-ST compared to the CST model[4] and/or to the DN-ST model being more sensitive to prolonged stimulus durations than visual transients[5,6] whereas the SEQ-SIM experiment is dominated by latter. **b-d** Simultaneous suppression levels vs CST_{opt} and CST_{fix} pRF parameters. For panels B-D (effective size, exponent, and tau pRF parameters), we first computed the median across pRFs of a visual area for each participant (similar to Fig 8), then we calculated the average of this median value across 7 participants. Pearson's correlation (*r*) is computed using individual participant data. *Filled colored circles*: CST_{opt} pRF parameters. *White circles with colored outline:* CST_{fix} pRF parameters. **b** Simultaneous suppression level vs median CST_{opt} pRF effective size. **c** Simultaneous suppression level vs CST_{opt} exponent (static nonlinearity). **d** Simultaneous suppression level vs time constant τ (tau). We find that the range of estimated spatiotemporal compression (exponent) in the CST_{opt} pRF model in the Kim *et al.* (2024) experiment is smaller, and compression is overall stronger than the spatiotemporal compression in the CST_{fix} pRF model, which was optimized to the SEQ-SIM data. *p*: p-value. *a.u.:* arbitrary units. **e** Average β-weights of sustained and transient channels for CST_{opt} pRF model. Beta weights are averaged first within a participant's visual area, then averaged across participants per visual area. *Colored bars:* Sustained channel. *White bars with colored outline:* Combined transient channel. *Dark gray dots:* Individual participant data for sustained transient channel. *Light gray dots:* Individual participant data for combined transient channel. Sustained and combined transient channels do not differ significantly from one another across visual areas. **f-i** Simultaneous suppression levels vs DN-ST pRF parameters**.** For all panels, we first computed the median across pRFs of a visual area for each participant (similar to Fig 8), then we calculated the average median value across participants. Pearson's correlation (*r*) is computed using individual participant data. **f** Simultaneous suppression level vs median DN-ST pRF effective size. **g** Simultaneous suppression level vs exponent (DN-ST *n*). **h** Simultaneous suppression level vs time constants. *Filled colored circles*: τ_1 parameter, time constants of the IRF. A smaller τ_1 results in an IRF that peaks earlier. *White circles with colored outline:* τ_2 parameter, time constant of the exponential decay. A smaller τ_2 results in quicker decay and stronger compression. **i** Simultaneous suppression level vs semi-saturation constant (σ_{DN}). A smaller semi-saturation results in stronger compression within the pRF. *ns*: not significant.

Supplementary Table 5. Log-likelihood ratio tests comparing goodness of fit of main linear mixed model (LMM) to three alternative linear mixed models. Likelihood ratio statistic (χ^2) corresponds to data in Supplementary Fig 6, where the main LMM uses a fixed intercept and slope for mean sequential amplitude as a function of stimulus condition and allowing for random participant intercept and slope per stimulus condition. Alternative LMM 1 uses a fixed intercept and slope for mean sequential amplitude and allowing one random slope per participant (N=10), except for IPS0/1 (N=4) and TO1/2 (N=8). Alternative LMM 2 uses a fixed intercept and slope for mean sequential amplitude as a function of stimulus condition and allowing one random slope per participant. Alternative LMM 3 uses a fixed intercept and slope for mean sequential amplitude as a function of stimulus condition and allowing a random slope per participant, per condition. *ΔDF:* difference in degrees of freedom between two models. *N:* number of observations (voxels).

Visual area	LMM 1	LMM 2	ΔDF	N	χ^2	*p*
V1	Main	Alternative 1	41	33324	1.4×10^4	10^{-5}
	Main	Alternative 2	35	33324	1.2×10^4	10^{-5}
	Main	Alternative 3	26	33324	5.2×10^3	10^{-5}
V2	Main	Alternative 1	41	33572	3.1×10^4	10^{-5}
	Main	Alternative 2	35	33572	2.0×10^4	10^{-5}
	Main	Alternative 3	26	33572	4.5×10^3	10^{-5}
V3	Main	Alternative 1	41	33872	3.6×10^4	10^{-5}
	Main	Alternative 2	35	33872	2.0×10^4	10^{-5}
	Main	Alternative 3	26	33872	9.5×10^3	10^{-5}
hV4	Main	Alternative 1	41	19188	2.2×10^4	10^{-5}
	Main	Alternative 2	35	19188	1.5×10^4	10^{-5}
	Main	Alternative 3	26	19188	4.8×10^3	10^{-5}
VO1/2	Main	Alternative 1	41	13484	2.0×10^4	10^{-5}
	Main	Alternative 2	35	13484	1.6×10^4	10^{-5}
	Main	Alternative 3	26	13484	8.8×10^3	10^{-5}
V3A/B	Main	Alternative 1	41	27488	3.3×10^4	10^{-5}
	Main	Alternative 2	35	27488	1.6×10^4	10^{-5}
	Main	Alternative 3	26	27488	7.3×10^3	10^{-5}
IPS0/1	Main	Alternative 1	41	3176	3.1×10^3	10^{-5}
	Main	Alternative 2	35	3176	2.5×10^3	10^{-5}
	Main	Alternative 3	26	3176	6.1×10^2	10^{-5}
LO1/2	Main	Alternative 1	41	35820	3.3×10^4	10^{-5}
	Main	Alternative 2	35	35820	1.5×10^4	10^{-5}
	Main	Alternative 3	26	35820	7.4×10^3	10^{-5}
TO1/2	Main	Alternative 1	41	6036	3.6×10^3	10^{-5}
	Main	Alternative 2	35	6036	2.0×10^3	10^{-5}
	Main	Alternative 3	26	6036	1.2×10^3	10^{-5}

References

1 Hessels, R. S., Niehorster, D. C., Kemner, C. & Hooge, I. T. C. Noise-robust fixation detection in eye movement data: Identification by two-means clustering (I2MC). *Behav Res Methods* **49**, 1802-1823 (2017). https://doi.org/10.3758/s13428-016-0822-1

2 Smeets, J. B. & Hooge, I. T. Nature of variability in saccades. *Journal of neurophysiology* **90**, 12-20 (2003). https://doi.org/10.1152/jn.01075.2002

3 Aqil, M., Knapen, T. & Dumoulin, S. O. Divisive normalization unifies disparate response signatures throughout the human visual hierarchy. *Proc Natl Acad Sci U S A* **118** (2021). https://doi.org/10.1073/pnas.2108713118

4 Kim, I., Kupers, E. R., Lerma-Usabiaga, G. & Grill-Spector, K. Characterizing spatiotemporal population receptive fields in human visual cortex with fMRI. *J Neurosci* **44**, e0803232023 (2024). https://doi.org/10.1523/JNEUROSCI.0803-23.2023

5 Zhou, J., Benson, N. C., Kay, K. N. & Winawer, J. Compressive Temporal Summation in Human Visual Cortex. *J Neurosci* **38**, 691-709 (2018). https://doi.org/10.1523/JNEUROSCI.1724-17.2017

6 Zhou, J., Benson, N. C., Kay, K. & Winawer, J. Predicting neuronal dynamics with a delayed gain control model. *PLoS computational biology* **15**, e1007484 (2019). https://doi.org/10.1371/journal.pcbi.1007484
