# Supplementary material for: Rethinking simultaneous suppression in visual cortex via compressive spatiotemporal population receptive fields
Source: Nat Commun. 2024 Aug 11;15:6885. doi: 10.1038/s41467-024-51243-7 (PMC11317513; doi:10.1038/s41467-024-51243-7)
Supplement: Supplementary file 3 — Reporting Summary [file 41467_2024_51243_MOESM3_ESM.pdf]

nature portfolio

Corresponding author(s): Eline Kupers

Last updated by author(s): Jun 29, 2024

Reporting Summary

Nature Portfolio wishes to improve the reproducibility of the work that we publish. This form provides structure for consistency and transparency in reporting. For further information on Nature Portfolio policies, see our Editorial Policies and the Editorial Policy Checklist.

Statistics

For all statistical analyses, confirm that the following items are present in the figure legend, table legend, main text, or Methods section.

n/a	Confirmed	
☐	☒	The exact sample size (*n*) for each experimental group/condition, given as a discrete number and unit of measurement
☐	☒	A statement on whether measurements were taken from distinct samples or whether the same sample was measured repeatedly
☐	☒	The statistical test(s) used AND whether they are one- or two-sided<br>*Only common tests should be described solely by name; describe more complex techniques in the Methods section.*
☐	☒	A description of all covariates tested
☐	☒	A description of any assumptions or corrections, such as tests of normality and adjustment for multiple comparisons
☐	☒	A full description of the statistical parameters including central tendency (e.g. means) or other basic estimates (e.g. regression coefficient) AND variation (e.g. standard deviation) or associated estimates of uncertainty (e.g. confidence intervals)
☐	☒	For null hypothesis testing, the test statistic (e.g. *F*, *t*, *r*) with confidence intervals, effect sizes, degrees of freedom and *P* value noted<br>*Give P values as exact values whenever suitable.*
☒	☐	For Bayesian analysis, information on the choice of priors and Markov chain Monte Carlo settings
☐	☒	For hierarchical and complex designs, identification of the appropriate level for tests and full reporting of outcomes
☐	☒	Estimates of effect sizes (e.g. Cohen's *d*, Pearson's *r*), indicating how they were calculated

Our web collection on statistics for biologists contains articles on many of the points above.

Software and code

Policy information about availability of computer code

Data collection	Stimuli presentation and response collection was implemented using MATLAB R2017b (MathWorks: https://www.mathworks.com/products/matlab.html) using PsychToolbox-3 (GitHub commit version 2e57cd0) and custom-written MATLAB code.
Data analysis	Whole-brain T1-weighted scans were aligned to the AC-PC line using SPM 12 (https://github.com/spm/spm12; latest version commit 3085dac) and auto-segmented with FreeSurfer v6.0 (https://surfer.nmr.mgh.harvard.edu/). Small manual corrections of segmentations were executed with ITK SNAP (v3.6.0; http://www.itksnap.org/pmwiki/pmwiki.php). fMRI data were preprocessed with the Vistasoft toolbox (https://github.com/vistalab/vistasoft; latest version commit 7f0102c), and aligned to whole brain anatomy using the alignvolumedata toolbox (https://github.com/cvnlab/alignvolumedata; latest version commit b513116). Subsequent behavioral, eye gaze, and fMRI data analyses were executed with custom-written code in MATLAB (R2020b) and publicly available at https://github.com/VPNL/simseqPRF (DOI:10.5281/zenodo.12658143) and https://github.com/VPNL/spatiotemporalPRFs (DOI:10.5281/zenodo.12658232). Eye gaze data were preprocessed with the I2MC toolbox (https://github.com/royhessels/I2MC; latest version commit f39948d).

For manuscripts utilizing custom algorithms or software that are central to the research but not yet described in published literature, software must be made available to editors and reviewers. We strongly encourage code deposition in a community repository (e.g. GitHub). See the Nature Portfolio guidelines for submitting code & software for further information.

Data

Policy information about availability of data

All manuscripts must include a data availability statement. This statement should provide the following information, where applicable:

- Accession codes, unique identifiers, or web links for publicly available datasets
- A description of any restrictions on data availability
- For clinical datasets or third party data, please ensure that the statement adheres to our policy

Minimally preprocessed behavioral, eye, and fMRI data are publicly available at https://osf.io/rpuhs/ (data for main paper figures) and https://osf.io/e83az/ (data for supplementary figures). Source data are available to create each data figure. In addition, for 7 participants who also participated in Kim et al. (2024), we downloaded pRF parameters (df_cv0_defaultHRF.mat) from the OSF storage page: https://osf.io/3gwhz/files/osfstorage.

Research involving human participants, their data, or biological material

Policy information about studies with human participants or human data. See also policy information about sex, gender (identity/presentation), and sexual orientation and race, ethnicity and racism.

Reporting on sex and gender	A roughly equal number of male and female subjects were recruited for participation in this research (participant's self-assigned sex: 6 female, 4 male). The investigators do not expect significant gender differences, thus no sex- or gender-based analyses were performed.
Reporting on race, ethnicity, or other socially relevant groupings	There was no exclusion in this study based on race or ethnicity, and investigators do not expect significant race or ethnicicty differences.
Population characteristics	No covariate-related population characteristics of the human participants were used in the experimental design or analysis.
Recruitment	Participants were recruited from the Stanford University community and participated in two separate fMRI scanning sessions: one retinotopy with whole brain anatomy scanning session and one simultaneous-vs-sequential (SEQ-SIM) visual paradigm scanning session. We do not expect biases in participant recruitment to meaningfully impact these results. Several participants were fMRI researchers (including all three authors) within the Stanford Psychology Department, which may have increased the data quality (insofar as it is dependent on participant motion and alertness) relative to a random population sample. Only authors KGS and ERK were aware of the specific hypotheses, we believe this awareness did not affect the results, as behavioral and eye fixation performance did not vary across conditions and was within range of other participants performance.
Ethics oversight	All study procedures were approved by the Stanford Internal Review Board on Human Subjects Research.

Note that full information on the approval of the study protocol must also be provided in the manuscript.

Field-specific reporting

Please select the one below that is the best fit for your research. If you are not sure, read the appropriate sections before making your selection.

☒ Life sciences ☐ Behavioural & social sciences ☐ Ecological, evolutionary & environmental sciences

For a reference copy of the document with all sections, see nature.com/documents/nr-reporting-summary-flat.pdf

Life sciences study design

All studies must disclose on these points even when the disclosure is negative.

Sample size	Population receptive fields (pRFs) are modeled within each participant and voxel independently. The sample size (number of participants) was determined from previous studies employing similar pRFs methodologies in the visual system which use data from a range of 5-28 participants (5 - Klein, Harvey, & Dumoulin, 2014 Neuron; 6 - Dumoulin & Wandell 2008 NeuroImage; 6 - Zhou et al. 2018 J Neurosci; 5 - Kay et al. 2013 J Neurophys; 12 - Stigliani et al. 2017 PNAS; 13 - Poltoraski et al. 2021 Nature Comms; 28 - Finzi et al. 2020 Nature Comms). Here we collected data from 11 participants. One participant was excluded due to excessive motion (see below) and we report data from 10 participants. The number of voxels per participant per area is specific to (i) the size of their independently-defined cortical visual areas, determined empirically from independent retinotopy data, and (2) coverage of these visual areas in corresponding data from the SEQ-SIM experiment. Overall, we obtained data in most participants' visual areas, except 6 participants who had insufficient coverage of IPS0/1 and 2 participants who had insufficient coverage of TO1/2, due to fewer slices in the SEQ-SIM experiment.
Data exclusions	One participant's data were excluded from the fMRI experiment due to excessive and abrupt head motion during scans and across scans (> 1 voxel, 2.4mm). For one participant, we could not collect eye gaze data in the SEQ-SIM experiment due to constraints in the mirror setup. From the 9 participants with continuously recorded eye gaze data, 4 participants were excluded due to excessive measurement noise or no data in >80% of time points within runs. Both criteria were established in advance.
Replication	Each voxel's data were analyzed independently for each visual area (~5000±1500 voxels per participant). This process was repeated independently in 10 participants. Voxel's data split-half reliability in the SEQ-SIM experiment was high: correlation of 0.70 to 0.96 across visual areas and participants. Voxel's data were fit using split-half run cross-validation procedure, separate for three pRF models (CST, compressive

	spatiotemporal summation; CSS, compressive spatial summation, and LSS, linear spatial summation). Results are consistent across voxels and individuals.
Randomization	All conditions were tested within-participants, within-voxels, so random allocation into groups was not necessary.
Blinding	Group allocation was not performed; thus, blinding was not necessary.

Reporting for specific materials, systems and methods

We require information from authors about some types of materials, experimental systems and methods used in many studies. Here, indicate whether each material, system or method listed is relevant to your study. If you are not sure if a list item applies to your research, read the appropriate section before selecting a response.

Materials & experimental systems

n/a	Involved in the study
☒	☐ Antibodies
☒	☐ Eukaryotic cell lines
☒	☐ Palaeontology and archaeology
☒	☐ Animals and other organisms
☒	☐ Clinical data
☒	☐ Dual use research of concern
☒	☐ Plants

Methods

n/a	Involved in the study
☒	☐ ChIP-seq
☒	☐ Flow cytometry
☐	☒ MRI-based neuroimaging

Magnetic resonance imaging

Experimental design

Design type	Retinotopy: Event-related (2-s per bar position). Sequential-vs-simultaneous: Block design (8s on/stimulus, 12s off/blank - mean luminance gray screen)
Design specifications	The main SEQ-SIM experiment had eight ~5.5-minute runs: 4 repeats of 2 runs (except for one participant, which had 3 repeats of 2 runs). The 2 runs contained 16 stimulus blocks, 4 repeats for each of the 8 conditions (2 sequence orders x 2 sizes x 2 timings). Each participant was assigned to a unique set of runs, where block order was pseudo-randomized across the two runs. Stimulus content (cropped squares from colorful cartoons) was updated for each participant's run, block, and trial.<br>The retinotopy experiment contained 4 repeated runs of ~3.4-minutes in which bar stimuli traversed across the visual in a circular aperture. Cartoon images inside the bar changed randomly at 8 Hz. The bar swept in 12 discrete steps, 2-s per bar position, for 4 orientations (0°, 45°, 90°, 135°) and 2 motion directions for each orientation.
Behavioral performance measures	Throughout the main SEQ-SIM experiment, participants fixated at the center of the screen, while performing a challenging RSVP letter 1-back detection task at fixation. Throughout the retinotopy experiment, participants fixated on a small colored dot at the center of the screen, while performing a color-change detection task of the dot (red to green, green to red). Performance was monitored via recorded button box presses, and fixation was monitored via eyetracking in the scanner.

Acquisition

Imaging type(s)	Functional and structural MRI
Field strength	3 Tesla
Sequence & imaging parameters	Structural MRI: T1-weighted, using a BRAVO pulse sequence (1 mm^3 isotropic, inversion time=450 ms, TE=2.912 ms, FA=12°), collected with a Nova 32-channel head coil. A T1-weighted inplane image (0.75x0.75x2.4 mm) was collected with the same coil and slice prescription as the functional scans to align functional and anatomical scans.<br>Functional MRI: T2*-sensitive gradient echo planar imaging sequence: 2.4 mm^3 isotropic, FoV=192 mm, TE=30 ms, FA=62°, TR=2000 ms for retinotopy and TR=1000 ms for SEQ-SIM experiment, collected with a Nova 16-channel head coil.
Area of acquisition	Structural MRI: Whole brain. Functional: EPI slice prescriptions were oblique, roughly perpendicular to the calcarine sulcus, acquiring data from occipital, parietal and temporal lobes. Retinotopy experiment had 28 slices, main SEQ-SIM experiment had 14 slices.

Diffusion MRI ☐ Used ☒ Not used

Preprocessing

Preprocessing software	Structural whole brain anatomy scans were aligned to the AC-PC line using SPM12 (https://github.com/spm/spm12) and

auto-segmented with FreeSurfer's recon-all algorithm (v6.0, http://surfer.nmr.mgh.harvard.edu/). Manual corrections of the FreeSurfer segmentation were done in ITKSNAP. Functional data were slice-time corrected, motion corrected, drift corrected, converted to percent signal change using the Vistasoft toolbox (https://github.com/vistalab/vistasoft). No spatial smoothing was applied. Participants' functional scans were aligned with the inplane to their whole brain anatomy scan, using a coarse, followed by a fine 3D rigid body alignment (6 DoF) using the alignvolumedata toolbox (https://github.com/cvnlab/alignvolumedata). The first 8 (SEQ-SIM) or 6 (retinotopy) volumes of each functional scan were removed to avoid data with unstable magnetization.

Normalization

No normalization was applied; all data were analyzed in the native brain space of each participant

Normalization template

The data were not normalized.

Noise and artifact removal

Between- and within-scan motion correction was applied, as well as high-pass filtering to remove fMRI drift.
Voxels with a split-half reliability <10% in the SEQ-SIM experiment, and pRF model goodness-of-fit (R^2) in the retinotopy data <20%.

Volume censoring

No volume censoring was done.

Statistical modeling & inference

Model type and settings

SEQ-SIM experiment: Independently for each of the 6 pRF models (3 main, 3 supplementary), we fitted each voxel's predicted time course to the observed time course with split-half cross-validated linear regression (ordinary least squares), resulting in a cross-validated coefficient of determination (cv-R^2) for each voxel. To quantify simultaneous suppression, we fitted a linear mixed model (LMM) to all participant's voxels within a visual area, using a maximum likelihood fitting method.
Retinotopy experiment: The CSS pRF model was fit to each voxel's average time course using 2-stage optimization (coarse grid-fit, followed by fine search-fit).

Effect(s) tested

The LMM predicted the average simultaneous BOLD response of each voxel as a function of the average sequential BOLD response, for each stimulus condition (fixed interaction effect), allowing for a random intercept and slope per participant and stimulus condition (random interaction effect). Differences in LMM regression slopes were tested with a two-way repeated measures ANOVA (factors: visual area and stimulus conditions across participants). If there was a main effect (p<0.05), we used Bonferroni-corrected post-hoc multiple comparison t-tests (two-sided) to evaluate differences between stimulus conditions and visual areas.
Differences in pRF model cv-R^2 were tested with a two-way repeated measures ANOVA across voxels of all participants and visual areas (factors: pRF model and visual area). If there was a main effect (p<0.05), we used Bonferroni-corrected post-hoc multiple comparison t-tests (two-sided) to evaluate differences between pRF models and visual areas.
Pearson's correlation r was used to quantify the relationship between participant slopes averaged across conditions and effective pRF size, exponent, time constant, or semi-saturation constant across visual areas.

Specify type of analysis: ☐ Whole brain ☐ ROI-based ☒ Both

Anatomical location(s)

Whole brain in the independent fMRI retinotopy data: All voxels' spatial pRF parameters were used to create visual field maps in the native brain space of each subject. These maps were used to draw borders that define visual areas using guidelines from the literature (V1/V2/V3 - Smith et al. 2001. Cerebral Cortex; hV4/VO1/VO2 - Witthoft et al. 2014 Cerebral cortex; LO1/LO2/TO1/TO2 - Amano et al. 2009. J Neurophys; V3A/V3B/IPS0/IPS1 - Swisher et al. 2007 J Neurosci).
ROI-based in the main SEQ-SIM experiment: For each subject and visual area, we created an ROI that selected voxels with pRFs centers within the circumference of big square stimuli: 8.82x8.82° square located 0.59° to 9.41° from display center in both x- and y-dimensions in lower left and upper right quadrant. From these voxels, we used those with corresponding data from the SEQ-SIM experiment.

Statistic type for inference

(See Eklund et al. 2016)

Voxel-wise. For each voxel, we report simultaneous suppression levels, pRF model parameters, cross-validated R^2. In addition, we report the average suppression level per stimulus condition, pRF size, exponent, time constant, semi-saturation constant across voxels within each visual area for each individual participant and across participants.

Correction

None.

Models & analysis

n/a	Involved in the study
☒	☐ Functional and/or effective connectivity
☒	☐ Graph analysis
☐	☒ Multivariate modeling or predictive analysis

Multivariate modeling and predictive analysis

We predict voxel's observed time course using six different image-computable, pRF encoding models. Spatial pRF parameters were independently estimated from each participant's retinotopy experiment using the CSS pRF model, resulting in a 2D Gaussian pRF with a center (x0, y0), standard deviation (σ) and exponent (n) parameter for each voxel. To predict voxel's responses in the SEQ-SIM experiment, we use the independent spatial pRF parameters to reconstruct LSS and CSS pRFs. The CST pRFs used the spatial pRF parameters from the retinotopy experiment and neural temporal IRFs with fixed parameters based on Stigliani et al. 2017. Only one CST pRF model parameter was optimized: the compressive spatiotemporal static power-law exponent using a grid-fit approach. In addition to the three main pRF models (LSS, CSS, CST), we tested three

additional pRF models to predict responses in the SEQ-SIM experiment. These modeling results are in the supplementary materials and include a center-surround Difference of Gaussians (DoG) model, a Delayed Normalization Spatiotemporal (DN-ST) model, and a CST model with optimized spatial and temporal parameters for each individual voxel (CST-opt). The DN-ST and CST-opt models follow the implementation by Kim et al. (2024) J Neurosci, using the publicly available optimized parameters with canonical HRF, by the published paper on OSF. The DoG model follows the implementation by Zuiderbaan et al. (2012) Journal of Vision and consists of of a center pRF subtracted by a surround pRF. The center pRF is identical to the LSS pRFs (using the same spatial parameters). For the surround pRF, we used the center pRF and scaled the size of each individual voxel within a given visual area by a fixed scale factor, based on the center:surround ratios reported in Aqil, Knapen, & Dumoulin (2021) PNAS.
